# The Galician MultiPic: a picture dataset that captures lexical variation

**DOI:** 10.3389/fpsyg.2025.1551000

**Published:** 2025-03-26

**Authors:** María Álvarez de la Granja, María Carmen Parafita Couto, Ana Rita Sá-Leite, Isabel Fraga, Jon Andoni Duñabeitia, Christos Pliatsikas, Montserrat Comesaña

**Affiliations:** ^1^Instituto da Lingua Galega, Universidade de Santiago de Compostela, Santiago de Compostela, Spain; ^2^Leiden University Center for Linguistics, Leiden University, Leiden, Netherlands; ^3^Language Variation and Textual Categorisation, Universidade de Vigo, Vigo, Spain; ^4^Seminar für Englische Philologie, Universität Göttingen, Göttingen, Germany; ^5^Instituto de Psicoloxía (IPsiUS), Universidade de Santiago de Compostela, Santiago de Compostela, Spain; ^6^Centro de Investigación Nebrija en Cognición (CINC), Universidad Nebrija, Madrid, Spain; ^7^School of Psychology and Clinical Language Sciences, University of Reading, Reading, United Kingdom; ^8^Human Cognition Lab, Centro de Investigação em Psicologia (CIPsi), School of Psychology, University of Minho, Braga, Portugal

**Keywords:** picture dataset, Galician, lexical variation, naming agreement, familiarity

## Introduction

In psycholinguistic research, careful selection and control of stimuli are essential for gaining insights into cognitive processes. Within this field, pictures often serve as stimuli, which requires the use of image databases to investigate linguistic, mnemonic, and visual perceptual phenomena in different populations (children without disabilities, adults, elderly people, illiterate, and brain-damage patients; see Soares et al., 2018 for more detail).

Although several image databases provide norms for variables such as naming agreement (the most common name assigned to a picture by individuals; Snodgrass and Vanderwart, 1980), conceptual familiarity (the frequency with which individuals encounter or think about the depicted object; Snodgrass and Vanderwart, 1980) and visual complexity (judgments regarding the number of lines, intricacies, and details in an image; Snodgrass and Vanderwart, 1980; see also Székely and Bates, 2000 for an objective measure of picture visual complexity), these norms are available in multiple languages but are often restricted to a limited set of black-and-white line drawings (less than 300). Notably, such black-and-white images have been found to elicit weaker recognition compared to colored pictures (Sanfeliu and Fernandez, 1996; Rossion and Pourtois, 2004). In recent decades, there has been an increased effort to develop colored image datasets in various languages. However, many of them consider small datasets (usually less than 500 pictures, but see Brodeur et al., 2014; and Krautz and Keuleers, 2022, for more extensive datasets) and/or use different normalization protocols that complicate the process of comparing data and planning and executing cross-linguistic experiments (Soares et al., 2018; Duñabeitia et al., 2022; Zhong et al., 2024 for overviews).

The Multilingual Picture (MultiPic) database (Duñabeitia et al., 2022) was designed to address the limitations of previous databases by providing researchers with norms for naming agreement and concept familiarity for a set of colored images (500), selected from an initial pool of 750 images (Duñabeitia et al., 2018). To date, this database spans thirty-three languages, including American English, Australian English, Basque, Belgium Dutch, British English, Cantonese, Catalan, Cypriot Greek, Czech, Finnish, French, German, Greek, Hebrew, Hungarian, Italian, Korean, Lebanese Arabic, Malay, Malaysian English, Mandarin Chinese, Netherlands Dutch, Norwegian, Polish, European Portuguese, Quebec French, Rioplatense Spanish, Russian, Serbian, Slovak, Spanish, Turkish, and Welsh. The images depict specific concepts and general knowledge items, and the same data collection and preprocessing protocols were consistently applied across all languages.

Expanding MultiPic to include additional languages, dialects, and language varieties worldwide would enable researchers to investigate lesser-studied languages beyond the predominant focus on English, facilitate direct cross-linguistic comparisons, and deepen our understanding of cognitive processes that are universal versus those that are language-specific. The primary objective of the present study was to norm MultiPic in Galician, a relatively under-researched language, enabling researchers to conduct studies with it. Galician is a Western Ibero-Romance language predominantly spoken in Galicia, an autonomous community in northwestern Spain, where it holds co-official status with Spanish.

Psycholinguistic studies on Galician are less prevalent than those on Spanish, Portuguese, Catalan, and Basque (see Comesaña and Sá-Leite, 2024). This paucity of research investigating the specific cognitive mechanisms involved in both the comprehension and production of Galician is likely attributable to several factors, including the language's recent standardization in the 1980s (Alonso Pintos, 2025) and the scarcity of databases that would allow for the careful selection of linguistic materials for various experiments. The development of these tools would reinvigorate research on Galician by ensuring that experimental outcomes accurately mirror core cognitive processes. Consequently, they would provide essential scientific evidence to inform public policies related to Galician. This language, which coexists with Spanish, presents a distinctive opportunity to examine psycholinguistic theories of language processing within bilingual contexts.

Although we generally adhere to the same data collection and preprocessing procedures described in the MultiPic database (Duñabeitia et al., 2022), several adaptations were necessary to accurately reflect Galician's linguistic reality. These modifications accounted for the linguistic diversity across the region, shaped by the contact between Galician and Spanish. For instance, because Galician remains subordinate to Spanish in many social contexts, speakers often incorporate Spanish words or adapted terms, with variations across different regions (cf. Rei-Doval, 2025). Thus, we considered the diverse linguistic varieties and regional differences within Galician, ensuring that the dataset represents the full spectrum of language use across different areas. This approach not only respects the sociolinguistic context of Galician but also allows for a more comprehensive understanding of the cognitive processes involved in bilingual language processing. That is, it will enable researchers to examine which cognitive processes are general and which are specific to different sociolinguistic contexts. Nevertheless, the experimental method, the preprocessing protocol, and the data structure are comprehensively detailed to provide researchers with the necessary framework for adapting the MultiPic to other languages with comparable sociolinguistic contexts.

In conclusion, MultiPic and the Galician MultiPic, in particular, serve as valuable tools that enable researchers to design studies in Galician and other languages, where the properties of the materials have been rigorously tested in parallel.

The complete dataset, including the data file, is publicly available in the following repositories: https://figshare.com/articles/dataset/Untitled_Item/19328939 and https://osf.io/ank4g/?view_only=43367d3dd27543b0aa66dfb8e71ce1fc.

## Method

### Participants

Participants were recruited over two months through social media and local newspaper advertisements. Their participation was voluntary. A total of 88 Galician speakers were initially recruited, surpassing the median sample size in the original MultiPic project (i.e., 80; Duñabeitia et al., 2022). Still, three were excluded for not following task instructions (e.g., responding in a language other than Galician or basing answers on familiarity with the picture instead of its name). From the remaining 85 participants (47 women, 34 men, four preferred not to disclose their sex; mean age = 42 [age range of 18 to 82], *SD* = 19.05), around 28% of participants were from O Grove, 8% from Santiago de Compostela, 8% from A Coruña, 6% from Vigo, and the rest from 24 different places. Even though all were speakers of Galician, in their daily lives, around 28% spoke only Galician, 32% spoke more Galician than Castilian Spanish, 18% spoke both languages equally, 16% spoke more Castilian Spanish than Galician, and 6% spoke only Castilian Spanish. More than half had a university degree.

### Materials

We used the 500 colored pictures from the MultiPic database representing common concrete concepts. These pictures were in PNG format with a 300 × 300 pixels resolution and were initially created by a local artist commissioned by the authors of the original study (Duñabeitia et al., 2018). The set of 500 elements depicted was the same as those used in Duñabeitia et al. (2022), consisting of a pictorial set of digital line drawings derived from a list of imageable and concrete Spanish words taken from EsPal (Duchon et al., 2013).

### Procedure

The Galician MultiPic norming followed the standardized protocol of the original MultiPic project. Instructions were provided in Galician. Sociolinguistic data, including age, gender, number of languages spoken fluently, and possession of a university degree, were collected. However, unlike other languages, the sociolinguistic data for Galician was gathered in greater detail to ensure an accurate understanding of the sociolinguistic reality of the Galician language. Thus, questions were added regarding place of birth and place of residence, age of acquisition of Galician and Spanish, language balance, educational level, and socioeconomic status.

First, participants were provided with a link and completed the tasks in the same order using the Gorilla Experiment Builder by typing their responses through a computer, tablet, or smartphone. However, fifteen elderly adults who were not computer literate provided their responses orally, which a team member transcribed. Considering this, all participants named each of the 500 randomly presented images, using no more than one word per concept. Then, they rated their familiarity with each concept on a 100-point scale, ranging from 0 (not familiar at all) to 100 (very familiar). If they did not know the name of an image, they could select the “?” button, which was recorded as an “I don't know” response. Before starting, participants completed two practice trials to familiarize themselves with the procedure. The experiment lasted approximately one hour, with breaks every 50 trials. Responses were coded to account for linguistic variations, including standard (i.e., the form accepted by the Real Academia Galega [Royal Galician Academy]) versus colloquial forms, dialectal differences, and influences from Spanish. To this end, and following preceding studies (Duñabeitia et al., 2018, 2022), a native speaker of Galician reviewed and corrected spelling errors while also standardizing responses by merging basic variants of the same names (e.g., hyphenated or pluralized forms).

### General description of the dataset

The spreadsheet “Galician MultiPic,” the detailed description of coding, and the raw data are available at the following link: https://osf.io/ank4g/?view_only=43367d3dd27543b0aa66dfb8e71ce1fc; also, in the Supplementary material section. The Galician MultiPic spreadsheet consists of three sheets: SUMMARY, H-STATISTIC and CODEBOOK. SUMMARY contains twelve columns. Column A, indicates the picture files (.png) nouns; Column B, the number of responses per picture (85); Column C, the H statistic; Column D, the modal response (i.e., the most common response, which is sometimes influenced by Spanish and does not have to align with the standard Galician form); Column E, the number of times the modal response was given; Column F, the most common standard Galician form provided; Column G, the number of times this most common standard Galician response was provided; Column H, number of other valid alternative responses; Column I, number of times the participants informed not knowing the response; Column J, number of times an idiosyncratic response (only one participant used that noun) was given; Column K and Column L, the mean and standard deviation for the familiarity scores, respectively; Column M, the English translation of each modal response in Column D. When the modal and the Galician standard form do not coincide, the latter is highlighted in yellow. Instances with two modal names are highlighted in green to reflect lexical variation within the dataset.

A version of the Galician MultiPic is also available at https://figshare.com/articles/dataset/Untitled_Item/19328939. However, only the data considering the Galician nouns are provided here, even when the Galician noun was not the modal name (which occurred with 52 nouns [highlighted in yellow in the dataset provided in OSF]). Thus, the Galician MultiPic at Figshare includes nine columns (from A to I) as occurs with the other 33 languages of MultiPic, which corresponds to the Language provided, the Code (number of the picture), the Number of Responses, the H statistic, the Modal Response, the Modal Response Percentage, the “I don't know” Response Percentage, the Idiosyncratic Response Percentage, and the Familiarity.

Sheet H-STATISTIC contains the calculation of the H-STATISTIC for each picture.

Sheet CODEBOOK contains a detailed description of the information collected in both the raw and cleaned data frames used for analyses.

## Results and discussion

Regarding the naming task, two measures were considered as in earlier MultiPic studies: the mean H statistic and the mean modal response percentage.^1^ These were analyzed, and the familiarity measures were recorded as well.

The most notable finding is that the data exhibits an averaged H statistic of 0.71 and a mean modal response percentage of 73.56%. As mentioned above, only 52 pictures out of 500 had one unique response. The H statistic for Galician is higher than the average for MultiPic across 33 other languages (0.55). Indeed, only 5 out of 33 languages (Malay, Lebanese, Korean, Mandarin, and Cantonese) have higher H statistic values than Galician, and only 2 (Mandarin and Cantonese) lower mean modal response percentages (73.28% and 59.17%, respectively). Interestingly, of the official languages in the Iberian Peninsula, including Basque, Catalan, Galician, Portuguese, and Castilian Spanish, Galician exhibits the highest H statistic and the lowest mean modal response percentage. In comparison, the H statistic and the mean modal response percentage for Basque are 0.66 and 82.94%, for Catalan 0.45 and 88.98%, for Portuguese 0.37 and 90.38%, and for Castilian Spanish 0.30 and 93%, respectively. Table 1 summarizes the norms for the 500 images of the MultiPic in each of the languages of the Iberian Peninsula. The relatively high mean H statistic and low mean modal response percentages obtained in the current dataset suggest a higher lexical variability when compared to most languages included in the MultiPic database and to the languages that coexist in the Iberian Peninsula.

**Table 1:** Distribution of each variable rating per language.

**Variables per language**	**Mean**	**SD**	**Min**	**Max**
Galician_H index	0.70	0.59	0.00	2.76
Catalan_H index	0.45	0.51	0.00	2.76
Basque _H index	0.66	0.58	0.00	2.59
Spanish_H index	0.30	0.40	0.00	1.66
European Portuguese_H index	0.37	0.48	0.00	2.35
Galician mean modal response %	73.56	0.59	0.00	2.76
Catalan mean modal response %	88.98	0.51	0.00	2.76
Basque mean modal response %	82.94	0.58	0.00	2.59
Spanish mean modal response %	92.96	0.40	0.00	1.66
European Portuguese mean modal response %	90.38	0.48	0.00	2.35
Galician_Familiarity	70.68	4.92	52.62	79.68
Catalan_Familiarity	75.46	8.84	45.67	90.94
Basque_Familiarity	74.56	7.69	43.39	86.76
European Portuguese_Familiarity	77.16	10.23	33.31	93.32

Correlation analyses on the H statistic and Familiarity values across languages of the Iberian Peninsula were conducted to validate individual dataset quality. We focus on comparative analyses in these languages because speakers share not only historical and linguistic connections but also cultural and educational influences that shape familiarity judgments. This is particularly relevant for Romance languages like Castilian Spanish, European Portuguese, Catalan, and Galician, which have significant lexical and structural similarities, as well as for Basque, which, despite being non-Romance coexists in the same sociolinguistic environment. While cross-linguistic correlations can occur even between typologically distant languages, as shown in previous studies, our focus here is on a more controlled linguistic and cultural space, allowing for a more precise interpretation of familiarity effects. See in the Table 2, the matrix of correlations.

**Table 2:** Matrix of correlations.

	**Familiarity_Cat**	**Familiarity_Basque**	**Familiarity_EP**	**Familiarity_Gal**	**H_Cat**	**H_Bas**	**H_EP**	**H_Gal**	**H_SP**
Familiarity_Cat	1	0.790^**^	0.797^**^	0.798^**^	−0.437^**^	−0.157^**^	−0.348^**^	−0.194^**^	−0.232^**^
Familiarity_Basque	0.790^**^	1	0.792^**^	0.791^**^	−0.294^**^	−0.252^**^	−0.330^**^	−0.121^**^	−0.270^**^
Familiarity_EP	0.797^**^	0.792^**^	1	0.838^**^	−0.218^**^	−00.057	−0.387^**^	−00.068	−0.202^**^
Familiarity_Gal	0.798^**^	0.791^**^	0.838^**^	1	−0.195^**^	−00.069	−0.270^**^	−0.104^*^	−0.162^**^
H_Cat	−0.437^**^	−0.294^**^	−0.218^**^	−0.195^**^	1	0.410^**^	0.523^**^	0.425^**^	0.589^**^
H_Bas	−0.157^**^	−0.252^**^	−00.057	−00.069	0.410^**^	1	0.393^**^	0.375^**^	0.428^**^
H_EP	−0.348^**^	−0.330^**^	−0.387^**^	−0.270^**^	0.523^**^	0.393^**^	1	0.274^**^	0.496^**^
H_Gal	−0.194^**^	−0.121^**^	−00.068	−0.104^*^	0.425^**^	0.375^**^	0.274^**^	1	0.429^**^
H_SP	−0.232^**^	−0.270^**^	−0.202^**^	−0.162^**^	0.589^**^	0.428^**^	0.496^**^	0.429^**^	1

* The correlation is significant at the 0.05 level (2 tails).

** The correlation is significant at the 0.01 level (2 tails).

A correlation analysis performed on the H statistic showed that all the Pearson pairwise correlation coefficients were significant at the *p* < 0.001 level, with *r* values ranging between 0.27 (Galician vs. European Portuguese) and 0.59 (Spanish vs. Catalan). The reason why *r* values between Galician and European Portuguese are the lowest despite their status as closely related languages with a shared medieval history as part of Galician-Portuguese, may be attributed to the distinct sociolinguistic contexts in which they have developed. These differing contexts have played a significant role in shaping lexical variation between the two languages. Note that Galician coexists with Castilian Spanish, a language of high prestige, which has led to the incorporation of numerous lexical borrowings from this language into Galician (see Dubert García, 2005). Furthermore, the establishment of an official written standard for Galician did not occur until 1980, highlighting the relatively recent process of linguistic standardization. In contrast, Portuguese is the main language in Portugal and does not coexist with another widely spoken language, except for Mirandese, which is used in the specific region of Miranda do Douro. Additionally, Portuguese has a long-established linguistic tradition, with its first grammar and dictionary dating back to the 16th century. Since these early efforts, a strong normative tradition has been maintained (Almeida, 2018).

Likewise, the correlation analyses performed on different familiarity scores obtained for each item in each language showed that all the Pearson pairwise correlation coefficients were significant at the *p* < 0.001 level, with *r* values ranging between 0.79 (Catalan vs. Basque) and 0.83 (Galician vs. European Portuguese).

Besides, a correlation analysis was conducted between the H statistic and Familiarity values in each official or co-official language from the Iberian Peninsula already tested in the MultiPic database. We found low to moderate negative and significant correlations in all of them. That is, the higher the values in familiarity, the lower the values in the H statistic, which makes sense as the higher the H statistic, the lower the name agreement. To be more precise, all Pearson pairwise correlation coefficients were significant at the *p* < 0.001 level, except for the Galician language (*p* = 0.02), with *r* values ranging between −0.10 (for the Galician) and −0.44 (for the Catalan). The smallest correlation was found for Galician. At first, we thought that this was probably because it has greater lexical variability than the other languages. Indeed, if we look at the second language from the Iberian Peninsula that has a high lexical variability (Basque), we can see that it also showed a small correlation value between H statistic and Familiarity (−0.25). However, when compared with other languages like Chinese or Malay that also have a great lexical variability we found high significant correlations (−0.49 and −0.90, respectively). Therefore, a more plausible explanation may lay on the fact that familiarity modulates agreement (and not the other way around). That is, if someone is not familiar (or that much familiar) with an object, they would be hesitant when naming it, and as a consequence, this would lead to lower agreement scores across participants. This would be true for all the languages. However, for Galician more variables than familiarity may be explaining this result such as the already mentioned coexistence with the Castilian language, the recent official written standard for Galician, which means that it is not perfectly implemented, and the desire of some people to reflect their dialectal variant. We recognize, however, that this is a tentative explanation that deserves further examination.

Each variable's inter-rater reliability was determined by calculating intraclass correlations (ICCs) via a two-way random consistency model. ICCs revealed acceptable reliability for H statistic (ICC = 0.78 [0.75, 0.81]), and an excellent reliability for familiarity (ICC = 0.92 [0.91, 0.93]).

Although all correlations were significant, findings underscore how social and regional factors, such as dialectal variation, hyper-Galician forms, and Spanish influence, shape lexical variation in Galician. A closer look at the responses given to each picture shows that some pictures had multiple interpretations that seem to reflect the variety of realities of the population, i.e., participants used nouns with different meanings (e.g., magnet vs. horseshoe), for example, by using different co-hyponyms or elements of the same semantic field (*figo* vs. *cebola* [fig vs. onion]), or, in some cases, by focusing on different elements or areas of the image (e.g., for the picture of a shoulder, participants used names like *costas, pel*, or *marrón*, i.e., back, skin, or brown in English). On the other hand, some pictures were named with synonyms, such as *xornal* and *periódico*, two different Galician words to name a newspaper. Importantly, in many cases, the noun participants used depended on the dialectal variety of their region (e.g., *vespa, avespa*, and *avéspora* for wasp). Also, in some cases, participants used “hyper-Galician” forms, i.e., linguistic forms that are mistakenly created when speakers attempt to use what they perceive as “correct” or “pure” Galician. People often make these mistakes to avoid the influence of Castilian Spanish, a tendency shaped by the unique context of language contact and the relatively late standardization of Galician in Galicia, as previously mentioned. For example, the correct Galician form for banana is *plátano*, but the hyper-Galician form *prátano* is sometimes used. Similarly, many examples of lexical transfers from Spanish can be observed, such as the Spanish word for cheese, *queso* or for elbow, *codo*. In fact, in more than 10% of the images (52 cases), the modal form is either the Spanish word (e.g., *destornillador* [screwdriver] or *vela* [candle]) or the adapted Spanish word (e.g., *xaula* [*cage* and *jaula* in English and Spanish, respectively] or *paiaso* [*clown* and *payaso* in English and Spanish, respectively]).

One may think, however, that the lexical variability observed in Galician is driven by the pictures rather than by the particularities of the language itself, as an anonymous reviewer pointed out. In other words, the same concepts consistently exhibit the lowest agreement across languages. This does not seem to be the case since when calculating the mean H statistic of the MultiPic database including all the languages tested thus far (33), this was 0.56 (standard deviation = 0.60) and the mean modal response percentage was 85.5% (standard deviation = 17.8). These values are similar to those provided in earlier studies with different sets of stimuli (e.g., Alario and Ferrand, 1999; Barry et al., 1997; Bonin et al., 2013; Dimitropoulou et al., 2009; Manoiloff et al., 2010; Rossion and Pourtois, 2004), and thus, as Duñabeitia et al. (2022) have already pointed out when comparing the data of 32 languages, “the relatively low mean H statistic and the high mean modal response percentages of the current dataset suggest high name agreement across items, languages, and varieties, validating the materials for their use in different kinds of experiments and tests” (p. 4).

Despite these contributions, challenges remain. The concentration of data from specific regions and the written modality of the experiment raise questions about representativeness and generalizability. Due to the relatively recent standardization of the Galician language, many speakers are unfamiliar with the correct spelling or form. This raises questions about the challenges of conducting this task in a written format. Nevertheless, this work underscores the importance of comprehensive planning and cultural awareness when developing multilingual resources. Moving forward, the Galician MultiPic offers valuable insights for cross-linguistic studies and bilingualism research, promoting recognition of linguistic diversity while providing a robust framework for future adaptations in other minority languages.

## Conclusions

The Galician adaptation of MultiPic fills a critical gap in psycholinguistic research by providing standardized norms for an underrepresented language. This resource highlights the rich lexical variation in Galician, shaped by regional diversity, bilingualism, and language contact with Spanish. Although issues like regional representation and modality (since MultiPic is a written rather than spoken task) remain, the Galician MultiPic is an essential tool for cross-linguistic studies and the exploration of bilingual cognitive processes, offering valuable insights for future research on minority language varieties.

## Data Availability

The datasets presented in this study can be found in online repositories. The names of the repository/repositories and accession number(s) can be found in the article/Supplementary material.

## Footnotes

^1^These two measures provide complementary pieces of information since while two concepts could have the same agreement percentage as measured by the mean modal response, H statistic values will differ on the basis of the number of alternative responses given to each of the concepts (see Dimitropoulou et al., 2009 for more detail).
