# Supplementary material for: The Galician MultiPic: a picture dataset that captures lexical variation
Source: Front Psychol. 2025 Mar 26;16:1551000. doi: 10.3389/fpsyg.2025.1551000 (PMC11979152; doi:10.3389/fpsyg.2025.1551000)
Supplement: Supplementary file 1 [file Supplementary_File_1.docx]

Supplementary_Material

# Coding: Decisions taken

**Adaptation to Regional and Linguistic Contexts:** Dialectal variations, hyper-Galician forms, and Spanish influences were systematically coded to capture the linguistic diversity and bilingual nature of the region.

**Unified Coding Rules:** Standardized guidelines were implemented for orthographic variations, gender and number forms, diminutives, and determiner usage, ensuring the dataset is consistent and comparable.

**Public Sharing:** The finalized dataset was made openly accessible to support psycholinguistic research and facilitate cross-linguistic studies.

These measures ensure that the Galician MultiPic serves as a reliable resource for researchers while faithfully representing the linguistic and cultural complexities of Galician.

If a person provided two or more responses (e.g., "hombreiro / costas" [shoulder/ back]), only the first one was recorded.

General Rules

- Articles or determiners preceding answers are excluded: "o altar" [the altar] is recorded as "altar."
- Answers with an initial capital letter are recorded in lowercase ("Libélula" becomes "libélula" [drangonfly]), unless they were proper nouns.
- Abbreviated forms are expanded ("carro combate" becomes "carro de combate" [tank]).
- Galician and Spanish forms are kept separate. Variants within each language are grouped according to frequency.

Grouping Variants

- Orthographic Variants: Grouped under the most frequent form ("volvoreta" and "bolboreta" under "bolboreta" [butterfly]), except when both forms are standard ("coala" and "koala" [koala] remain separate).
  - If orthographic differences reflect Galician vs. Spanish, forms are kept separate ("harpa / arpa" [harp]).
- Phonetic/Morphological Variants: Dialectal differences ("man" and "mao/mau" [hand]) or vowel alternations ("cortina" and "curtina" [curtain]) are grouped unless both are standard ("sarabia" and "saraiba" [hail] remain separate ).
- Hypergalician Forms: Incorrect Galician forms ("pruma" [feather], "prátano" [banana]) are grouped under the standard ("pluma," "plátano").
- Incorrect accent marks: Forms with incorrect accent marks ("carcere" [jail]) are grouped under the correct version ("cárcere"). Differences due to Galician vs. Spanish ("aureola / auréola" [aureole]) are kept separate.
- Typos: Typographical errors ("rato" and "raato" [mouse]) are grouped under the correct form ("rato").

Singular/Plural Forms: Grouped under the most frequent number ("pezón" and "pezóns" [nipple(s)] under "pezón"; "peito" and "peitos" [breast(s)] under "peitos").

Gender Variants: Grouped under the more frequent gender ("elefanta" under "elefante" [elephant]). If responses are equally frequent, they default to the masculine form ("pelirroxo" and "pelirroxa" under "pelirroxo" [readhear/ginger]).

Evaluative Affixes: Diminutives or augmentatives ("gatiño" [little cat]) are grouped under the base form ("gato" [cat]). Lexicalized forms ("serrón" [handsaw], "queixelo" [jaw]) remain separate.

General vs. Specific Terms: If a generic term and a more specific term refer to the same item ("bata" and "bata de casa" [robe]), they are grouped under the more general term ("bata").

Complex vs. Simple Expressions: Simplified or reduced forms are grouped with their more frequent counterparts ("barra" and "barra de pan" [loaf of bread] under "barra de pan").

Shortened vs. Full Forms: Shortened forms ("tatu") are grouped under the full term ("tatuaxe" [tattoo]) unless both are in the dictionary ("bici" and "bicicleta" [bicycle] remain separate).

Adaptations: Spanish words adapted to Galician are grouped under the more frequent version ("carretera" and "carreteira" under "carretera" [road]). If the adaptation is more frequent, both forms remain separate ("espantapájaros" and "espantapaxaros" [scarecrow]).

Hybrid Forms: When forms can be linked to either Galician or Spanish (e.g., "sirea"), they are grouped under the more frequent version ("serea" [mermaid]).

Other Exceptions: Unless otherwise noted, all remaining cases not previously covered are kept separate.

"Don’t Know" Responses: Answers like "imaxe" [image], "icono" [icon], "interrogante" [question], "non sei" [I don’t know] or "non..." [no] are recorded as "DON'T KNOW."

# H-Index calculation

The H index is a statistical value that reflects the level of agreement across participants in a set of responses (Shannon & Weaver, 1949). It is calculated using the following formula:

H=pi log21/pi

Where:

- *p*_i_​ is the proportion (probability) of each response.
- log_2_(1/*p*_i_) is the base-2 logarithm of the inverse probability.

For example, for the first picture (PICTURE_1.png):

1. Compute the Proportion (*p*_i_​):
   - For "rato": *p* = 65/84 = 0.7738
   - For "furón": *p* = 15/84 = 0.1786
   - For "ratón": *p* = 4/84 = 0.0476
2. Compute log_2_(1/*p*):
   - This represents the base-2 logarithm of the inverse probability (log_2_(1/*p*_i_)).
   - For "rato": log_2_(1/0.7738) = 0.3699
   - For "furón": log_2_(1/0.1786) = 2.4854
   - For "ratón": log_2_(1/0.0476) = 4.3923
3. Compute *p*_i_ × log_2_(1/*p*_i_):
   - Multiply each proportion by the corresponding log_2_(1/*p*_i_).
   - For "rato": 0.7738×0.3699 = 0.28630
   - For "furón": 0.1786×2.4854 = 0.44380
   - For "ratón": 0.0476×4.3923 = 0.20920
4. Sum the Values:

H = 0.2863+0.4438+0.2092 = 0.93930

Thus, the H index is approximately 0.9393. This value reflects the uncertainty or variability of the responses, with a value of 0 indicating no variability (only one unique valid response was given).

**3. Instructions in Galician**

Benvida/o a esta tarefa!

Durante esta sesión amosarase unha longa listaxe de debuxos e terás que responder algunhas preguntas sobre cada un deles.

Antes de comezar, terás que contestar algunhas cuestións básicas sobre ti.

Empecemos! Preme o botón para comezar.

Por favor, escribe un alias para cubrir o cuestionario (non poñas o teu nome e apelidos reais):

INFORMACIÓN RELEVANTE SOBRE O CONSENTIMENTO INFORMADO

- A participación neste estudo é completamente voluntaria.
- A participación neste estudo non afectará á/ao participante desde un punto de vista médico ou académico.
- Este estudo é só para fins de investigación e os datos recollidos non se utilizarán para o diagnóstico clínico.
- As respostas a este cuestionario suporán información anónima respecto da persoa participante.
- O uso dos datos das/dos participantes para o estudo final dependerá das/dos investigadoras/es, e é posible que non se inclúan algúns dos datos proporcionados por cuestións técnicas ou outras circunstancias específicas.
- A/O participante poderá retirar o seu consentimento en calquera momento.
- Calquera pregunta sobre o estudo, a técnica utilizada ou o propósito pode facerse en calquera momento (mariacarmen.parafita@uvigo.gal)
- As investigadoras/es ou responsables informarán á/ao participante de todo o anterior ou de calquera outro dato ou incidencia destacable.
- As investigadoras/es ou responsables solicitarán o seu consentimento previo ao inicio do estudo.

Preme neste cadro para dar o teu consentimento.

Top of Form

Selecciona a opción que se axeita mellor a ti:

 son un home
 son unha muller
 prefiro non dicilo

Selecciona a túa idade (en anos):

| 18 | 18 | 99 |
| --- | --- | --- |

Lugar de nacemento (concello):

                                                                                                                                                                                                                                                                                                                                                                                                                                                                                                                                                                                                                                                   

Lugar actual de residencia (concello):

                                                                                                                                                                                                                                                                                                                                                                                                                                                                                                                                                                                                                                                   

No caso de que vivises noutros lugares por períodos longos de tempo, indícao:
Por ex.

Lugar: A Habana, Cuba
Datas:1975-93

Lugar: Zas, A Coruña
Datas 1993-99

Desde cando falas galego?

 Desde que tiña dous anos ou incluso antes
 Desde que tiña catro anos ou incluso antes
 Desde a escola primaria
 Desde a escola secundaria
 Aprendín a falar galego de adulto

Desde cando falas castelán?

 Desde que tiña dous anos ou incluso antes
 Desde que tiña catro anos ou incluso antes
 Desde a escola primaria
 Desde a escola secundaria
 Aprendín a falar castelán de adulto

Normalmente falas...

 Só galego
 Só castelán
 Máis galego ca castelán
 Máis castelán ca galego
 As dúas linguas por igual

Cantas linguas, incluída a túa lingua nativa, falas con fluidez?

                     

Tes un grao universitario?

 Non
 Si
 Son estudante universitaria/o

Cal é o teu nivel máis alto de educación?

 Sen estudos
 Primarios incompletos
 Primarios completos (Graduado escolar)
 Secundarios incompletos
 Secundarios completos (BUP – COU - ESO - Bacharelato - Ciclo Formativo de Grado Medio)
 Diplomatura o Ciclo Formativo de Grado Superior
 Licenciatura ou Grao e/ou Doutoramento

Imaxina que estas escaleiras representan os distintos estratos sociais. Na parte superior da escaleira están as persoas que teñen máis cartos, máis educación e mellores traballos. Na parte inferior están as persoas que se atopan na peor situación social, con menos cartos, menos estudos, peores traballos ou mesmo desempregados. Selecciona o chanzo que mellor represente onde pensas que estás na escaleira. 1 representa o chanzo máis baixo e 10 o máis alto.


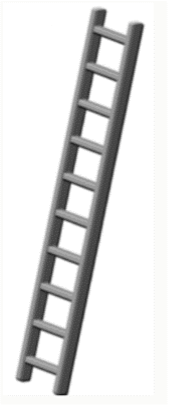


Selecciona:

 10
 9
 8
 7
 6
 5
 4
 3
 2
 1

1. **Instructions in English**

Welcome to this task!

Durante esta sesión amosarase unha longa listaxe de debuxos e terás que responder algunhas preguntas sobre cada un deles.

Antes de comezar, terás que contestar algunhas cuestións básicas sobre ti.

Let´s get started! Press the button to start.

Please, enter an alias to fill out the questionnaire (do not put your real name and surname):

RELEVANT INFORMATION ABOUT INFORMED CONSENT

• Participation in this study is completely voluntary.

• Participation in this study will not affect the participant from a medical or academic point of view.

• This study is for research purposes only, and the data collected will not be used for clinical diagnosis.

• The answers to this questionnaire will involve anonymous information about the participating person.

• The use of participant data for the final study will depend on the researchers, and some of the data provided may not be included due to technical issues or other specific circumstances.

• The participant may withdraw their consent at any time.

• Any question about the study, the technique used, or the purpose can be asked at any time ([mariacarmen.parafita@uvigo.gal](mailto:mariacarmen.parafita@uvigo.gal))

• The researchers or managers will inform the participant of all the above or any other noteworthy data or incident.

• The researchers or managers will request their consent before the start of the study.

During this session, you will be shown a long list of drawings, and you will have to answer a few questions about each of them.

Before you begin, you must answer some basic questions about yourself.

Click on this box to give your consent.

Select the option that best suits you:

 I am a man
 I am a woman
 I´d rather not to say it

Select your age (in years):

| 18 | 18 | 99 |
| --- | --- | --- |

Place of birth (municipality):

                                                                                                                                                                                                                                                                                             

Current place of residence (municipality):

                                                                                                                                                                                                                                                                                                         

If you lived in other places for long periods of time, please indicate:

For example:

Place: Havana, Cuba
Data:1975-93

Place: Zas, A Coruña
Data: 1993-99

Since when do you speak Galician?

 Since I was two years old or even earlier
 Since I was four years old or even earlier
 Since primary school
 Since secondary school
 I learned to speak Galician as an adult

Since when do you speak Spanish?

 Since I was four years old or even earlier
 Since primary school
 Since secondary school
 I learned to speack Spanish as an adult

You usually talk

 Galician only
 Spanish only
 More Galician than Spanish
 More Spanish than Galician
 Both languages equally

How many languages, including your native language, do you speak fluently?

                     

Do you have a college degree?

 No
 Yes
 I am a college student

What is your highest level of education?

 Without studies
 Incomplete primary
 Primary (Graduate school)
 Incomplete Secondary
 Secondary (BUP – COU - ESO – High School – Certificate of Intermediate Studies)
 Diploma or Certificate of Advanced studies
 Degree or BA/BS and/or PhD

Imagine that these stairs represent the different social strata. At the top of the ladder are people with more money, education, and better jobs. At the bottom are the people who are in the worst social situation, with less money, less education, worse jobs, or even unemployed. Select the rung that best represents your position on the ladder. 1 represents the lowest step, and 10 is the highest.

*
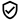
*


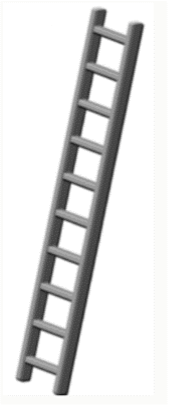


Select:

 10
 9
 8
 7
 6
 5
 4
 3
 2
 1

# Supplementary Figures and Tables

For more information on Supplementary Material and details on the different file types accepted, please see [here](https://www.frontiersin.org/guidelines/author-guidelines#supplementary-material).

## Supplementary Figures

**
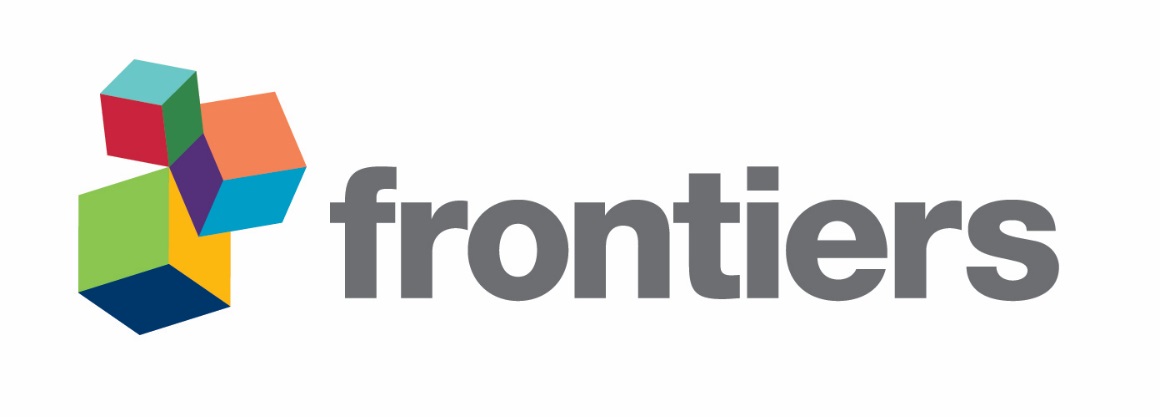
**

**Supplementary Figure 1.** The figure legends are required to have the same font as the main text, 12 point normal Times New Roman, single spaced. Please use a single paragraph for each legend and prepare the figures keeping in mind the PDF layout.
